# Heads or tails: L1 insertion-associated 5' homopolymeric sequences

**DOI:** 10.1186/1759-8753-1-7

**Published:** 2010-02-01

**Authors:** Thomas J Meyer, Deepa Srikanta, Erin M Conlin, Mark A Batzer

**Affiliations:** 1 Department of Biological Sciences, Biological Computation and Visualization Center, Louisiana State University, 202 Life Sciences Bldg, Baton Rouge, LA 70803, USA

## Abstract

**Background:**

L1s are one of the most successful autonomous mobile elements in primate genomes. These elements comprise as much as 17% of primate genomes with the majority of insertions occurring via target primed reverse transcription (TPRT). Twin priming, a variant of TPRT, can result in unusual DNA sequence architecture. These insertions appear to be inverted, truncated L1s flanked by target site duplications.

**Results:**

We report on loci with sequence architecture consistent with variants of the twin priming mechanism and introduce dual priming, a mechanism that could generate similar sequence characteristics. These insertions take the form of truncated L1s with hallmarks of classical TPRT insertions but having a poly(T) simple repeat at the 5' end of the insertion. We identified loci using computational analyses of the human, chimpanzee, orangutan, rhesus macaque and marmoset genomes. Insertion site characteristics for all putative loci were experimentally verified.

**Conclusions:**

The 39 loci that passed our computational and experimental screens probably represent inversion-deletion events which resulted in a 5' inverted poly(A) tail. Based on our observations of these loci and their local sequence properties, we conclude that they most probably represent twin priming events with unusually short non-inverted portions. We postulate that dual priming could, theoretically, produce the same patterns. The resulting homopolymeric stretches associated with these insertion events may promote genomic instability and create potential target sites for future retrotransposition events.

## Background

Retrotransposons, mobile elements that move via a 'copy and paste' mechanism, called retrotransposition, are ubiquitous in primate genomes [1,2]. L1s, members of the long interspersed element (LINE) family of non-long terminal repeat (LTR) retrotransposons, which comprise as much as ~17% of primate genomes, are present in copy numbers of approximately 520,000 and have actively molded primate genomic architecture for the last 65 million years [3-5]. During their mobilization, they generate insertions containing L1 sequence and, in some cases, transduced sequence and deletion of adjacent genomic sequence [6-9]. Long after insertion, however, L1s can serve as sites of non-allelic homologous recombination, resulting in the loss, gain and inversion of genetic material [10,11]. In these ways, L1s have been shown to disrupt genes, cause disease states and contribute to the expansion and contraction of the genome [12-14].

**Figure 1:**
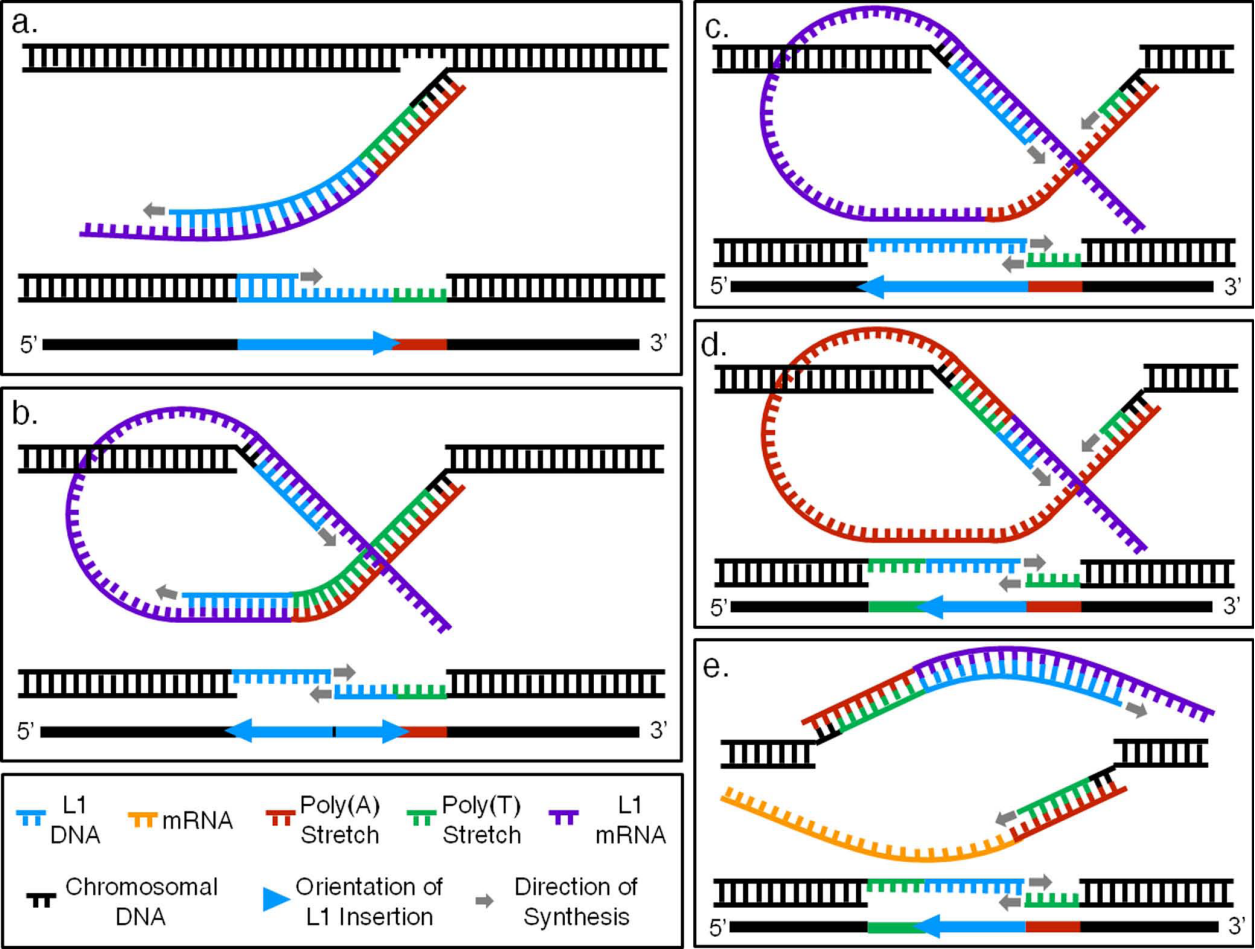
**Classical target primed reverse transcription (TPRT), twin priming, variants of twin priming and dual priming mechanisms**. (a) A schematic of classical TPRT. The poly(A) tail of an L1 mRNA anneals to the target site created by L1 endonuclease. L1 reverse transcription (RT) primes at the target site and synthesizes the bottom-strand cDNA. A subsequent second-strand nick and synthesis results in an L1 insertion with a 3' poly(A) flanked by TSDs. (b) Twin priming. In this variant of TPRT, after the second-strand nick, a site internal to the mRNA anneals to the top strand overhang. A second RT molecule primes at this site, generating an inverted L1 cDNA. (c) This twin priming variant involves the disengagement of the first RT before reaching the end of the poly(A) tail, resulting in an insertion with a 5' poly(T) stretch, but lacking a 3' poly(A) tail. Like classical twin priming, this mechanism results in an inverted L1 structure. (d) A second twin priming variant creates an insertion with both a 3' poly(A) tail and a 5' poly(T) stretch. The first RT falls off before reaching the end of the poly(A) tail. (e) Dual priming. Classical TPRT involving the first mRNA begins on the first strand. After the second strand nick, a second mRNA anneals to the second strand and undergoes classical TPRT. Note that this panel is rotated 180° relative to the orientation of all other panels. This is done to show that the resulting insertion will appear the same to computational filters as the above twin priming variant.

These autonomous retrotransposons contain a 5' untranslated region (UTR) with an RNA polymerase II promoter, two open reading frames (ORFs), and a 3' UTR encompassing a poly(A) tail; full-length L1s are ~6 kb long [15]. ORF1 encodes an RNA-binding protein with nucleic acid chaperone activity and ORF2 encodes both a reverse transcriptase (RT) and an endonuclease (EN) [16-19]. The L1 EN and RT are integral to an insertion process, termed target primed reverse transcription (TPRT), used by L1s to insert *de novo *copies of themselves into their host genomes [20] (Figure 1a). Non-autonomous retrotransposons, like *Alu *and SVA elements, use the L1 retrotransposon enzymatic machinery for their own mobilization via TPRT [21,22].

The classical TPRT mechanism involves a single nick on the bottom strand at a loosely-preferred cleavage motif (foe example, 5'-TTTT/A-3') by the EN, leaving a free 3' hydroxyl group at the nick site. The L1 mRNA then anneals to the nick using its poly(A) tail and L1 RT uses this mRNA as a template for reverse transcription beginning at the free 3' hydroxyl group. Top strand cleavage, integration of the cDNA, and synthesis of a top strand complement to the cDNA complete the insertion, leaving the structural hallmarks of classical TPRT: intact target site duplications (TSDs), a typical EN cleavage site motif, and a variable length poly(A) tail [17,20,23,24]. While full-length L1s are ~6 kb in length, many L1 insertions are 5' truncated (averaging ~900 bp in length) and no longer able to actively retrotranspose [13,15,24,25]. Anomalies observed in TPRT-inserted copies have led to the proposal of variant mechanisms, such as internal and twin priming, that account for non-standard sequence architecture for TPRT-inserted elements (Figure 1b) [9,26-29]. Recent studies have shown that insertions using twin priming lead to new retrogene formation, limit L1 expansion and cause genome instability [26,30].

**Figure 2:**
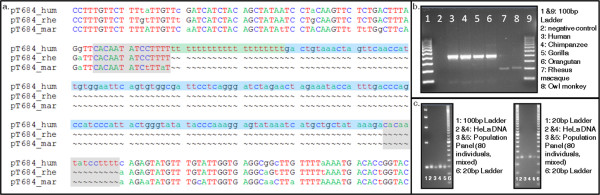
**Investigation of candidate loci and variations within the homopolymeric stretches**. (a) A triple alignment of pT684 to two outgroup species, the rhesus macaque and the common marmoset. The TSDs are highlighted in grey, the poly(T) stretch in green, and the L1 is highlighted in blue. (b) A gel chromatograph of polymerase chain reaction products depicting an insertion present in humans, chimpanzees, gorillas and orangutans, but absent in rhesus macaque and owl monkey. (c) Internal primers were designed around the poly(T) stretches for all human-specific loci; two loci are shown here. For each locus, HeLa DNA and a mixture of the DNA of 80 human individuals was run out on a 4% agarose gel with 100 bp and 20 bp ladders. No within-species variation in poly(T) length was observed.

A recent human genome-wide analysis led to the discovery of homopolymeric thymine (poly(T)) stretches just upstream of truncated L1 insertions [29]. Intrigued by these homopolymeric stretches associated with loci having many hallmarks of classical TPRT, we performed a computational analysis of the available assembled primate genomes, experimentally verified the resulting candidates and describe the characteristics typical of these loci (Figure 2a). We refer to, and examine, all candidate loci as poly(T) stretches 5' of sense-oriented L1s, although the mechanisms we propose, that may account for this appearance, suggest that these poly(T) stretches are, in fact, the poly(A) tail of a complex retrotransposon insertion involving inverted L1 sequence. Here we report 39 examples and, as mechanisms to account for the observed structures, we propose two variants of twin priming that result in an inversion-deletion of the L1 sequence and introduce dual priming, a mechanism involving the priming of both bottom- and top-strand nicks by two different mRNAs (Figure 1c-e). The resulting homopolymeric stretches generated by these events may act as sites of genomic instability and as potential targets for future retrotransposon insertions.

## Results and discussion

### Investigation of homopolymeric stretches at the 5' ends of mobile elements

In order to determine whether the homopolymeric stretches of nucleotides at the 5' ends of insertions were particular to poly(T)s, we first investigated the most complete assembled primate genome available at the time of analysis, build hg18 of the human genome. Our computational filters returned only those loci for which a simple repeat was found immediately upstream of an L1, *Alu *or SVA element. Poly(A)s were found to be the most numerous followed by poly(T)s (527 and 170 loci, respectively) (Table 1). Poly(C) and poly(G) loci, on the other hand, were relatively rare (1 and 5 loci, respectively). Close inspection of these loci revealed that only poly(T) stretches were found between the 5' TSD and the 5' end of a sense-oriented retrotransposon insertion. The numerous poly(A)s were found to be the poly(A) tails of insertions interrupted by the insertion of another element, and were not restricted to the space between the 5' TSD and the 5' end of an element. None of the poly(C)s or poly(G)s were found within the TSDs and at the 5' ends of retrotransposon insertions. Furthermore, none of the loci associated with *Alu *or SVA insertions in the human genome were found to match our criteria. Hence, we restricted further analyses in other primate genomes to the investigation of poly(T)s found between the 5' TSD and the 5' end of an L1 insertion. The mechanism or mechanisms responsible appear to involve only the creation of homopolymeric thymine stretches upstream of L1s. These observations implicate the autonomous machinery associated with L1s as necessary components in the insertion process.

**Table 1:** Computationally-derived loci from assembled primate genomes.

	H	C	O	Rh	Combined
**poly(T)**	169	183	290	276	918
**poly(A)**	522	646	809	909	2886
**poly(C)**	1	4	0	0	5
**Poly(G)**	4	9	8	1	22
**Loci**	696	842	1107	1186	3831

Computational filters were used to detect loci based on the proximity (<20 bp) of simple repeats to the 5' end of an L1.

### Characterization of candidate loci

Of the 918 loci, our computational filters produced, 54 passed our manual inspection, 39 of which also passed wet-bench verification (Table 2). These loci represent a total of ~37.9 kb of inserted sequence. The insertions ranged from 99 to 4697 bp in total length, with an average length of 971 bp. Insertion-mediated deletions were virtually non-existent, with a total of only 5 bp deleted relative to the pre-insertion sequence. In 17 of the 39 loci, the insertion locus contained only the poly(T) stretch and the truncated L1. The remaining 22 loci included some non-candidate L1 sequence inserted along with the candidate L1 and poly(T) stretch. This extra sequence ranged in size from 4 bp to 2263 bp, with an average of 319 bp, and contributed a total of ~12.5 kb of inserted non-candidate L1 sequence. The proposed mechanisms described below allow for the addition of other mRNA sequence during the TPRT event and may account for the observed non-candidate L1 sequence in these loci. For example, recent studies have described retrogene formation through the twin priming mechanism, though analysis of our non-candidate L1 sequence did not find evidence of this [26,30]. We believe that none of our loci resulted in transduced sequence, and the extra sequence inserted with our candidate L1s very possibly represents 'filler' DNA [30,31]. The TSDs ranged in length from 7 bp to 20 bp, with an average of 14 bp. The 5' poly(T) stretches ranged from 14 bp to 39 bp, with an average of 23 bp. These poly(T) stretches were subject to nucleotide substitutions, as expected with any sequence, but appeared relatively well-conserved as non-(T) nucleotides contributed only 3.6% of the total length of all poly(T)s (33 of 911 bp). A comparison of poly(T) lengths among orthologs revealed evidence for some post-insertional modification (Figure 2b). However, a further inspection of our human-specific loci, through gel electrophoresis and Sanger cycle-sequencing, showed no variation between individuals (Figure 2c). The candidate L1s ranged from 61 bp to 2399 bp, with an average length of ~615 bp (Table 2). None of our candidate loci were intragenic and they appear to have inserted randomly throughout the genome. While we find no full-length L1s in our dataset, the limited number of loci and likely biases of our proposed mechanisms against full-length insertions make this unsurprising.

**Table 2:** Candidate loci and insertion site characteristics

Locus	Coordinates	TSD	L1 bp ins	Non L1 seq	Poly(T)	Lineage
pT44	chr1:80856707-80866878	14	653	487	23	H
pT79	chr11:104048005-104058372	11	1960	1599	25	H
pT415	chr3:181306257-181316602	16	339	0	29	H
pT512	chr5:83637882-83648058	18	1406	1165	26	H
pT546	chr6:69193896-69204810	16	927	0	23	H

pT439	chr3:62933035-62943400	9	1116	758	21	HC
pT684*	chr7:117312394-117332534	14	157	0	20	HC

pT1313	chr3:147198235-147208351	17	369	257	23	C
pT1350	chr6:55186000-55196486	15	470	14	32	C
pT1362	chr7:89293399-89303535	9	2399	2263	26	C
pT1389	chr9:97107198-97117833	14	691	68	20	C

pT43	chr1:72796354-72806494	16	112	0	24	HCG
pT1223	chr1:59822991-59833038	17	1022	4	20	HCG
pT1279	chr18:44020257-44030483	15	2015	1813	18	HCG

pT144	chr13:101611291-101621562	8	1145	866	22	HCGO
pT145	chr13:104133249-104143781	11	529	0	22	HCGO
pT325	chr2:101586549-101596728	16	181	0	27	HCGO
pT424	chr3:199260458-199270665	14	734	536	18	HCGO
pT458	chr4:172846531-172856775	17	234	4	19	HCGO
pT1309	chr2b:226703516-226713749	13	228	0	24	HCGO
pT1448	chr11:86639999-86650182	9	182	0	30	HCGO

pT1404	chr1:181059564-181069827	15	913	654	23	O
pT1416	chr1:7600379-7611178	13	791	0	21	O
pT1431	chr11:100399372-100409835	10	456	12	21	O
pT1465	chr13:57849422-57859574	15	175	27	17	O
pT1535	chr2a:44695595-44705774	15	165	0	23	O
pT1538	chr2a:70854440-70864821	11	377	0	23	O
pT1554	chr2b:66983758-66993962	17	174	20	21	O

pT1709	chr10:72142313-72152683	13	379	0	21	Rh
pT1712	chr11:100852416-100862528	20	105	0	34	Rh
pT1743	chr13:4175512-4185626	13	98	0	19	Rh
pT1785	chr17:40754900-40765052	15	1390	1244	34	Rh
pT1790	chr17:68109266-68119556	14	294	0	39	Rh
pT1798	chr18:71236237-71246385	7	252	118	19	Rh
pT1834	chr3:159608718-159618930	17	457	257	14	Rh
pT1846	chr3:75648970-75659040	14	61	8	16	Rh
pT1855	chr4:153605855-153616110	17	252	0	21	Rh
pT1896	chr6:3989032-3999144	15	97	0	36	Rh

pT1796	chr18:36523812-36534192	9	665	294	17	HCGORh

* Indicates locus previously described in [29]

Ins, insertion, Seq, sequence; TSD, target site duplication

### Alignment to ancestral full-length consensus sequences and subfamily contributions

**Figure 3:**
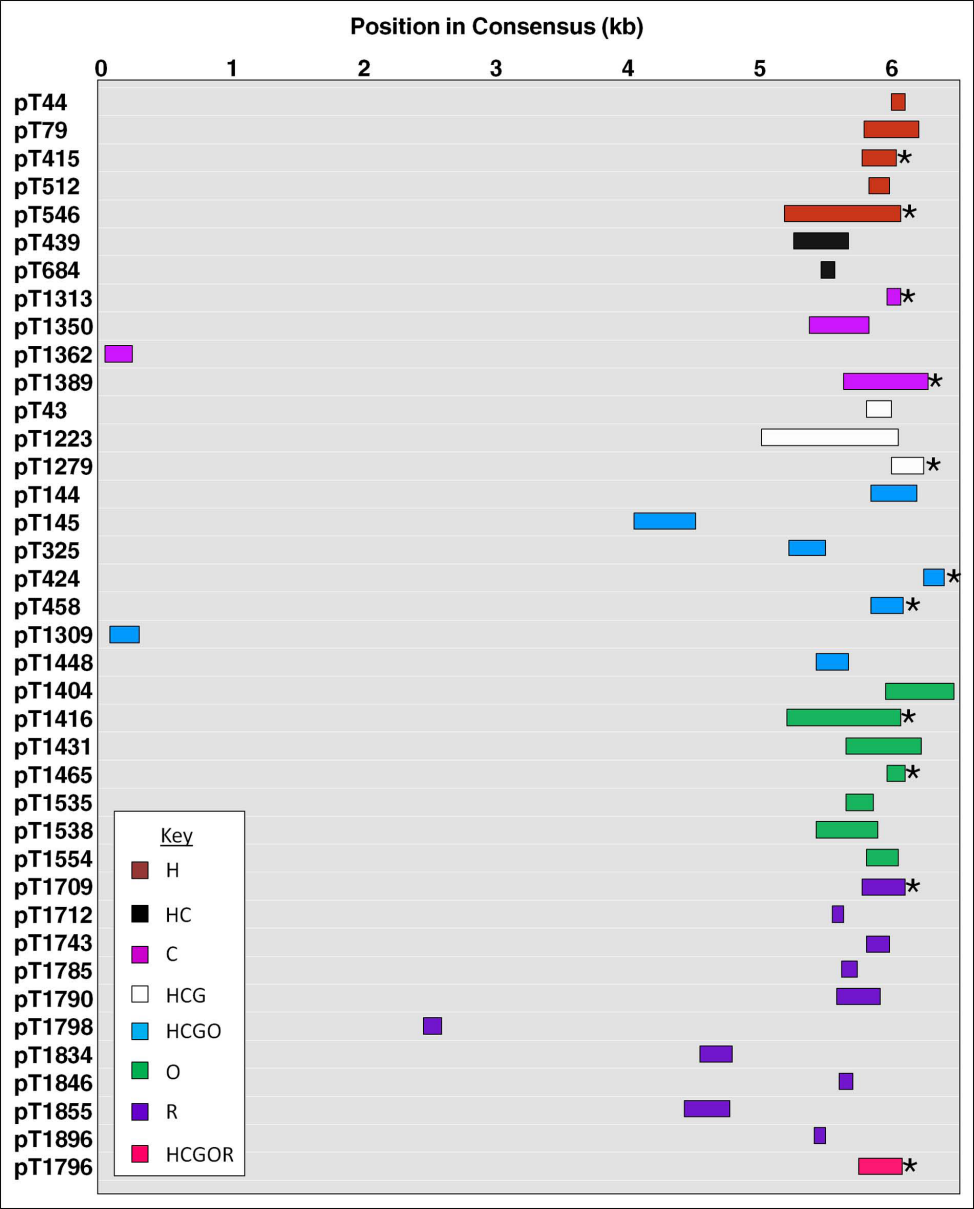
**Alignment of candidate L1s to their L1 consensus sequences**. Schematic of the position of each candidate L1 when aligned against an L1 consensus sequence. Stars indicate that the 3' end of the locus aligns to a portion of the poly(A) tail in the consensus. Loci are color-coded to indicate in which species each was found.

Most L1s in the genome are 5' truncated, and L1 3' truncation is relatively rare [4,15,25,26]. In all but two of our loci, the L1s were found to have substantial 5' truncations, aligning close to or at the 3' end of their corresponding consensus sequence. The two exceptions to this trend are pT1309 and pT1362, which are heavily 3' truncated and align near, but not at, the beginning of their respective consensus sequences. In 11 of the 37 heavily 5' truncated loci, a short but identifiable section of the poly(A) tail is present. The remaining 26 loci, while aligning near the 3' end of the consensus, do not reach the poly(A) tail, and are therefore 3' truncated as well (Figure 3).

The pre-insertion structure of each locus was determined through triple-alignment with its orthologs in two outgroups that did not contain the insertion (Figure 2a). Two New World monkeys (Haplorrhines), the common marmoset and owl monkey, were used as outgroups when investigating Catarrhine-specific loci (those shared between humans, chimpanzees, gorillas, orangutans and Old World monkeys). Haplorrhine-specific loci, however, were not investigated in this study and, though loci shared between the Catarrhines and Haplorrhines were recovered by our computational filters (data not shown), these were excluded from our analyses because a suitable sequenced outgroup lacking the insertions was not available. Our findings that these loci occur throughout the region of the primate tree investigated, in both lineage-specific instances and as shared insertions dating from before the divergence of Haplorhines and Catarrhines (~40 mya) [1,32], suggest that whatever mechanism or mechanisms cause this distinct sequence architecture has occurred in primate lineages from ancient to recent times.

### Analysis of the junctions within poly(T) loci: microhomology and target site analyses

**Figure 4:**
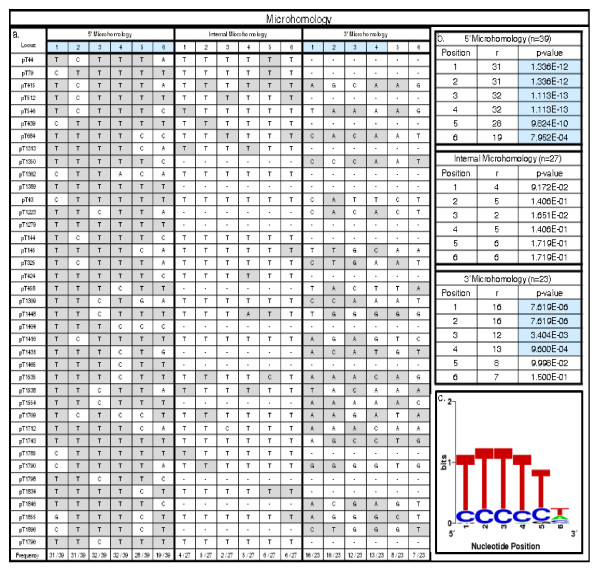
**Microhomology and comparison of insertion site characteristics of each locus**. (a) An analysis of the microhomology of the six nucleotides adjacent to each insertion junction (with '1' indicating the nucleotide closest to the insert) to the corresponding sequence in the putative mRNA. Exclusion of a junction from analysis is indicated by a (-) and positions where microhomology is found are shaded grey. Those positions at which significant microhomology were found are highlighted in blue. (b) A binomial distribution analysis of the 6 bp at each junction revealed significant microhomology at both the 3' and 5' junctions of the insertions. No significant microhomology was found at the internal junction. *P*-values highlighted in blue are significant at *p *< 0.001. (c) A WebLogo analysis of the 6 bp found at the 3'junction. The logo supports our finding of microhomology at this junction, and is consistent with the expected motif at the L1 endonuclease cleavage site.

Inspection of microhomology at the junctions between TSDs and inserts is useful in distinguishing between competing mechanisms [8,26,33,34]. We analysed the microhomology of three junctions within each locus: the points where the TSDs met the insertion, both 5' and 3', as well as the internal point where the poly(T) stretch met the L1 insertion (Figure 4a). For the 5' junctions, we reverse complimented our sequences, which allowed us to examine our loci as if the candidate L1s had been inserted in the antisense fashion. We found significant microhomology (*p*-value < 0.001) at positions one through four of the 3' insertion junction and at all six of the positions analysed at the 5' insertion junction. There was no significant microhomology found at the internal junction between the poly(T) stretch and the truncated L1 (Figure 4a, b). To verify the position of the internal junction and reduce any errors attributable to RepeatMasker, we aligned the reverse-complemented poly(T) stretch and 50 bp downstream to an L1 consensus sequence. If RepeatMasker had miscalled the end of the L1 element, we should have been able to align some portion of this reverse complemented stretch to the 3' end of the L1 consensus. As we were unable to find any alignment between these sequences and the 3' end of the consensus, we concluded that our internal junctions were correctly identified. We further suggest that the internal junction was repaired using non-homologous end-joining (NHEJ), rather than finding microhomologous points.

**Figure 5:**
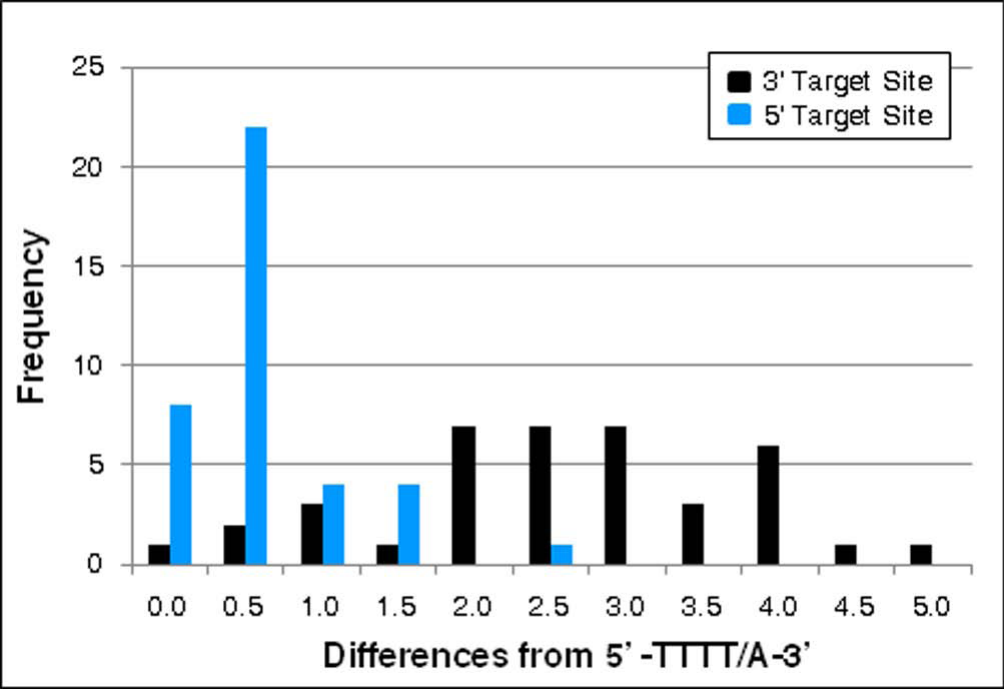
**L1 endonuclease (EN) cleavage site analyses at the 5' and 3' junctions**. For both the 5' and 3' target sites of each locus, the last four nucleotides of the target site and first nucleotide of the flanking sequence were compared to the canonical L1 EN cleavage motif (5'-TTTT/A-3'). To investigate the possibility that the candidate L1s were inserted in the antisense orientation, the 5' target site was reverse complemented and analysed. The black bars show the frequency of each divergence value at the 3' target site among our 39 loci, while the blue bars show values for the 5' target sites. The 3' target sites show more divergence from the typical EN cleavage motif than the 5' target site.

A comparison of the target sites of our loci to the canonical TPRT L1 EN cleavage site (5'-TTTT/A-3') was also performed in order to determine whether L1 EN was involved in the production of these loci. When our loci were oriented such that our candidate L1s were in the sense orientation, the 3' junctions did not closely match the expected pattern. However, when this analysis was performed on the reverse complement of the 5' junction, we found almost no deviation from the canonical EN cleavage site (Figure 5). This finding is emphasized by a sequence logo of the 5' ends of our TSDs showing a strong preference for (T)s at the first five positions of that junction (Figure 4c) [35]. This is consistent with a process involving L1 enzymatic machinery and suggests that our candidate L1s were actually inserted in the antisense orientation and that the poly(T) stretch is a portion of the poly(A) tail of the insertion.

### Elimination of possible mechanisms that could account for observed sequence architecture

Several possible insertion mechanism variants were considered as potentially leading to the distinct sequence architecture observed at these loci. First, and most simply, these loci could be the result of assembly errors in the published genomes. Rigorous inspection of sequences across all available primate genomes, as well as polymerase chain reaction (PCR) verification and sequencing eliminated assembly error as a possible explanation. Homopolymeric stretches are known to expand and contract as a result of post-insertion modification (for example, strand slippage) [36-38] and this may be advanced to explain the poly(T) stretches associated with our loci. We did find evidence of such modifications when we sequenced loci after PCR amplification on primate panels while investigating between-species variation. However, the variation did not exceed 10 bp. In the most extreme case of this type of modification (pT458), an ortholog to a 19 bp poly(T) stretch in the human was found to be only 9 bp in the chimpanzee after sequencing. Most loci in our dataset, however, showed less variation among orthologs. Also, when we analysed the variation in poly(T) lengths within the human species for each human-specific locus in the data set, no differences in size among individuals were found (Figure 2c). In addition, post-insertion modification would be expected to act on other homopolymeric stretches (poly(A)s, poly(C)s, and poly(G)s) with equal frequency. Furthermore, stretches associated with L1s should be just as likely as those associated with *Alu *and SVA elements to expand in this manner. Our data indicate that this phenomenon is restricted to poly(T) stretches and we have only recovered loci matching the described sequence architecture from candidates involving L1s. Therefore, while we acknowledge that homopolymeric stretches may undergo expansion and contraction, we reject it as an explanation accounting for the full length of our poly(T)s and the specific characteristics of our loci.

After eliminating assembly errors and post-insertional modification as possible mechanisms for this phenomenon, we searched for known mechanisms by which these structures may be formed. Non-template base addition, RNA editing and the activity of terminal transferase have all been shown to add extra sequence onto the 5' ends of L1 insertions [39-41]. However, these mechanisms result in relatively short stretches of added nucleotides and this is inconsistent with the large poly(T) stretches seen in this study. The RT of HIV has been shown to undergo a reiterative mode of DNA synthesis resulting in repetitive sequences not present in the template of a range of lengths inclusive of those we see in the poly(T) stretches of our loci [42]. While theoretically possible, this activity has not been reported in association with any L1 RT. Additionally, this mechanism requires specific motifs in the template at the site of the reiterative synthesis and we found no significant microhomology at our internal junctions (Figure 4a, b) [42].

This led us to speculate about the possible involvement of cryptic promoter activity to explain the observed patterns [43]. A cryptic promoter immediately upstream to a pre-existing stretch of poly(T)s, which was itself upstream of an L1, could result in a 5' stretch of poly(T)s in a *de novo *insertion. Alternatively, a cryptic antisense promoter located 3' to an L1 locus could be hypothesized to generate an antisense L1 mRNA including some 3' flanking sequence at its 5' end. Once reverse transcribed, this mRNA would produce a *de novo *insertion corresponding to the sequence architecture we see in our loci. In this scenario, the poly(A) tail added to the mRNA prior to insertion would appear to be a 5' poly(T) stretch if the candidate L1 is viewed in the sense orientation. This would also account for why we see non-candidate L1 sequence at the 3' ends of 22 of our 39 loci. However, this mechanism should also be easily identifiable by locating the original sequence, including the downstream antisense promoter, elsewhere in the genome. In all 22 cases involving non-candidate L1 sequence, original loci were not able to be reliably located, and we therefore conclude that cryptic promotion, while possible, is inconsistent with our observations.

### Twin priming events resulting in inverted poly(A) tails

Subsequently, we considered twin priming, a mechanism that did not at first appear to be consistent with the patterns we observed in our loci. This mechanism results in L1 inversions accompanied by internal deletions to the L1 sequence [9,26,44]. In this mechanism, the L1 mRNA anneals using its poly(A) tail to the bottom strand EN nick site and an RT primes at this location and begins to synthesize the L1 cDNA exactly as in classical TPRT (Figure 1a). However, once the top strand is nicked, generating a 3' overhang, this model proposes that a position internal to the mRNA may anneal to the overhang, allowing a second RT molecule to prime and begin synthesizing cDNA in the antisense orientation on the top strand. The resulting twin priming insertion is characterized by TSDs bounding two inverted fragments of the same L1 and containing an internal deletion of the L1 sequence (Figure 1b). An assumption of the twin priming mechanism is that the second strand nick must occur before first strand reverse transcription is completed [26,30].

In light of our microhomology results, it seems likely that the poly(T) stretches at the 5' ends of our L1s are, in fact, the poly(A) tails of the L1 insertions as reverse transcribed by the first RT molecule of a twin priming event. To remain consistent with our observed sequence architecture, the first RT molecule must cease reverse transcription prior to the end of the poly(A) tail of the mRNA, while the second, top-strand RT molecule of the twin priming event synthesizes a portion of the L1. The resulting insertion would take the form of an antisense L1 followed by a sense-oriented poly(A) tail, the anti-parallel strand of which would present a poly(T) stretch at the 5' end of an L1 (Figure 2a). Our candidates would not have been detected in previous studies of twin priming because these studies were specifically focusing on loci containing two inverted L1 fragments within TSDs. Below, we discuss variations of the standard twin priming model that may more accurately portray mechanisms that would result in the observed patterns.

The target site analyses and microhomology results we obtained implicate a variant of TPRT as the mechanism generating these loci. We found significant microhomology at the 5' end of the poly(T) stretch and the 3' end of the L1 insertion. Interestingly, it is not the 3' target site that closely resembles the canonical L1 EN cleavage site, but the complementary sequence of the 5' target site nearest the stretch of poly(T)s. As described above, our analysis of the reverse-complemented sequence adjacent to the poly(T) stretch recovered no evidence of inverted L1 sequence at this junction. While previous twin priming studies found some microhomology at the internal junction, this was usually less than that found at the target site, and in some cases, no microhomology was found [26,30]. One explanation that may account for this appearance involves the poly(A) tail of the element being reverse transcribed, but assumes that this first RT disengages prior to exiting the tail and entering the L1 sequence proper. The other priming event, occurring internally on the mRNA, then synthesizes a portion of the L1 cDNA. When viewed with the candidate L1 in the sense orientation, the poly(A) tail is reverse complimented, forming a stretch of poly(T)s located 5' to the L1 (Figure 1c). To determine if a short portion of non-inverted L1 sequence was found after the poly(T) stretches, a simple check involving an alignment of the reverse complement of the poly(T) stretch and following 50 bp of our insertions to an L1 consensus could find no match to the 3' end of the consensus.

Eleven loci include short portions of a poly(A) tail at the 3' end of the sense-oriented L1 sequence (Figure 3). For these loci, we propose a twin priming variant in which the poly(A) tail of the mRNA was long enough to be the site not only of the initial priming event on the bottom strand, but also the site of the internal priming event on the top strand (Figure 1d). These two twin priming variants adequately explain all of our observed loci except those that align close to the 5' end of their consensus sequence (pT1309 and pT1362). We conclude, therefore, that twin priming variants involving one transcription event that does not leave the poly(A) tail could provide a potential explanation of the observed sequence morphology.

### Dual priming

We speculate that another mechanism, which we term 'dual priming', could result in the same sequence characteristics described above. This mechanism involves two mRNAs annealing to the two nick sites. The first mRNA anneals to the bottom strand and undergoes normal TPRT, generating a sense-oriented L1 cDNA. After the top strand nick occurs, a second mRNA molecule may anneal with its poly(A) tail to this top strand overhang, allowing a second RT molecule to prime and generate a cDNA in the antisense orientation on the top strand (Figure 1e). If this top strand RT molecule disengages prior to exiting the poly(A) tail of its mRNA, it would create the same sequence architecture predicted by the twin priming variants. We are unable to distinguish between the twin priming and dual priming mechanisms given the current data set. The computational filters used generated loci in which the gap between the poly(T) stretch and candidate L1 was = 20 bp, limiting the size of potentially identifiable non-inverted mobile element sequence, making its identification via BLAT or RepeatMasker impossible at the time of analysis. The authors hope future studies will validate the dual priming mechanism.

We found no microhomology at the internal junction of our loci; this aspect is less consistent with the pattern of twin priming insertions observed in previous studies [26,30]. If dual priming occurs, microhomology should also be expected at the internal junction between the two cDNAs. This lack of microhomology at our internal junctions suggests that it is unnecessary for either of these mechanisms. A recent study of the effects of the NHEJ (non-homologous end joining) pathway on LINE retrotransposition implicated these proteins in the joining of the 5' ends of TPRT-mediated insertions [45]. In a twin or dual priming mechanism, the analogous position to the 5' end of a classical TPRT-mediated insertion is the internal junction. It was also indicated that NHEJ involvement resulted in truncation, a characteristic shared by all 39 of our loci. We therefore speculate that repair at this junction may, at least sometimes, be facilitated by NHEJ pathways instead of microhomology-dependent pathways [26,45,46].

## Conclusions

A growing body of research has shown that L1 insertions have shaped the genomic landscape across the Mammalia [2,47]. Recent insights into variations in integration pathways have added a deeper level of understanding of the dynamism lent by mobile elements to the genome. Our loci appear to have inserted via a mechanism or mechanisms that make use of TPRT but result in non-standard insertion structures. Through a combination of computational data mining, PCR analysis and Sanger cycle-sequencing, we have characterized a set of 39 truncated L1s with a poly(T) stretch at the 5' end of the insertion. Our analyses of the lineages show that this phenomenon is not specific to a particular lineage or period of retrotransposon expansion. These features are largely consistent with twin or dual priming, but the lack of microhomology at the internal junction may suggest a role for NHEJ proteins in the repair process. The homopolymeric stretches resulting from these insertion events could act as sites of instability, contributing to genomic fluidity [48-50]. This study further illustrates the impact L1s have on their host genomes and adds to the diversity of insertion mechanisms.

## Methods

### Computational and manual inspection of candidate loci

We first downloaded the RepeatMasker output for the hg18 assembly using the University of California atSanta Cruz (UCSC) Table Browser utility [51,52]. Next, we used in-house Perl scripts to find all loci at which RepeatMasker identified a simple repeat (poly(A), poly(T), poly(C) or poly(G)) within 20 bp upstream of either an L1, SVA or *Alu *element, resulting in 3831 computationally-derived loci. The anti-sense alternative of each possibility was also accounted for in the scripts. The nibFrag utility bundled with the BLAT software package [53] provided sequence for each locus, including 5000 bp flanking sequences both up- and downstream of the locus. We used a local installation of RepeatMasker to scan our loci on the sensitive setting in order to provide more accurate calls for repeats in these sequences [52]. After screening the human genome, it was determined that no locus involving an upstream poly(A), poly(C) or Poly(G) signal was found to match our search criteria. In addition, these loci would most likely make up an insignificant number of targets in the non-human genomes. Thus, poly(A)s, poly(C)s and poly(G)s were excluded from further analysis. *Alu *and SVA elements were also not found to be involved in loci matching our search criteria and were eliminated from the screenings of the chimpanzee, orangutan and rhesus macaque genomes. The common marmoset genome (calJac1) was not used as a source of loci because, at the time of publication, this genome was only available in contig form as opposed to the fully assembled primate genomes. However, it was used during the manual inspection of loci. In all, this computational filtering process produced a set of loci from the four assembled primate genomes (human (hg18), chimpanzee (panTro2), orangutan (ponAbe2) and rhesus macaque (rheMac2)) numbering 918 (Table 1).

These computationally-derived loci with added flanking sequence were then used to query the possible outgroup genomes (human, chimpanzee, orangutan, rhesus macaque and common marmoset) using the BLAT software suite [53]. A triple alignment of each locus, with two outgroups lacking the insertion, was created in order to analyse the local pre-insertion and post-insertion sequence architecture (Supplemental Data). In these triple alignments, we scanned for the presence of TSDs and for any target-site deletions present in the pre-insertion sequence, but absent following the L1 insertion. Additionally, we identified repeated loci that had been mined from different genomes, but which were orthologous, making sure to only count each locus once, regardless of how many species by which it was shared. We kept for further analysis all loci, regardless of the age of the associated L1 element, as long as the integration events had easily reconstructed pre-insertion sequence architecture.

We chose to retain for experimental validation the 54 loci that matched the following four criteria: presence of TSDs = 6 bp in length, verifiable pre-insertion sequence structure in at least one other primate genome, presence of a poly(T) stretch touching the 5' TSD and within 20 bp of the 5' end of the candidate L1 insertion. All analyses were performed by orienting the candidate L1 in the sense-orientation, unless otherwise specified.

### PCR amplification and sequencing to authenticate candidate loci

We PCR-amplified all loci on a panel of primate genomes, and sequenced all ambiguous loci and 20% of the locus set obtained from each genome. We designed primers for each locus using the Primer3 utility [54] and performed PCR in 25 μl reactions using 15 ng-25 ng genomic DNA, 0.28 μM primer, 200 μM dNTPs in 50 mM KCl, 1.5 mM MgCl_2_, 10 mM Tris-HCl (pH 8.4) and 2.5 units *Taq *DNA polymerase. Thermocycler programs were as follows: 95°C for 2 min (1 cycle), [95°C for 30 sec, optimal annealing temperature for 30 sec, 72°C for 2 min] (35 cycles), 72°C for 10 min (1 cycle). PCR products were visualized on 1%-2% agarose gels stained with ethidium bromide. For PCR fragments with expected lengths larger than 1.5 kb, ExTaq™ (Takara) was used according to the manufacturer's specified protocol. All loci were amplified from the following genomic DNAs: *Homo sapiens *(HeLa; cell line ATCC CCL-2); *Pan troglodytes *(common chimpanzee 'Clint'; cell line Coriell Cell Repositories NS06006B); *Gorilla gorilla *(Western lowland gorilla; cell line Coriell Cell Repositories AG05251); *Pongo pygmaeus *(orangutan; cell line Coriell Cell Repositories GM04272A); *Macaca mulatta *(rhesus macaque; cell line Coriell Cell Repositories NG07109); and *Aotus trivirgatus *(Owl monkey; cell line ATCC CRL-1556). In some cases, primate panel amplification did not work with the orangutan genomic DNA and we achieved a successful amplification using two alternative orangutan individuals, *Pongo pygmaeus *(Bornean orangutan; cell line Coriell Cell Repositories AG05252) and *Pongo abelii *(Sumatran orangutan; cell line Coriell Cell Repositories 12256).

Each human-specific locus was analysed in order to determine whether the candidate insertion was polymorphic within a panel of 80 individuals (20 African Americans, 20 Asians, 20 Europeans and 20 South Americans). These loci were further investigated in order to determine the length and within-species variability of their poly(T) sequences using internal primers and a pooled DNA sample comprised of the 80 individuals used above. PCR amplicons of each poly(T) sequence and <50 bp flanking in each direction were size fractionated on 4% high resolution agarose gels to check for length differences within humans. Primer sequences are available from the publications section of the Batzer laboratory website http://batzerlab.lsu.edu (Supplemental Data).

Outgroup loci were sequenced directly from the PCR amplicons after cleanup using Wizard^® ^gel purification kits (Promega Corporation) or ExoSAP-IT^® ^(USB Corporation). The poly(T) loci could not be sequenced directly from PCR products and were cloned into vectors using the TOPO TA (fragments <2 kb) cloning kit (Invitrogen). Following cloning, two to four colonies were randomly selected for colony PCR. Those colonies that appeared to contain the insert were then mini-prepped using the manufacturer's protocol (5PRIME). Sequencing results were obtained using an ABI3130XL automated DNA sequencer and analysed using BioEdit http://www.mbio.ncsu.edu/BioEdit/page2.html and the SeqMan and EditSeq utilities from the DNAStar^® ^V.5 software package. Close inspection of the flanking sequence and the results of PCR were used to confirm the pre-insertion sequence for each locus from a minimum of one outgroup genome. Sequences generated in this study have been deposited in GenBank under Accession Nos GQ477185-GQ477273.

### Microhomology and L1 endonuclease cleavage site analyses

The 6 bp of the 3' TSD closest to the insert were compared to the corresponding sequence at those positions in an alignment of each candidate L1 fragment to the L1 consensus in the manner described in Sen *et al*. [34]. The 3' junctions of some loci were excluded from analysis if a non-candidate L1 sequence was included in the insert. At the internal junction between the poly(T) stretch and the 5' end of the candidate L1, the first 6 bp of the L1 were compared to the last 6 bp of the poly(T) and the internal junction of a locus was excluded if any non-candidate L1 sequence was found between the poly(T) stretch and candidate L1.

EN cleavage site analysis of the 3' target site of each locus for similarity to the preferred L1 EN cleavage motif (5'-TTTT/A-3') was carried out by comparing this motif to the first four bases of the reverse complemented TSD and the first base of the flanking sequence. Differences in base composition were scored with transitions given a weight of 0.5 and transversions given a weight of 1.0 [8,33]. The frequency of divergence from the L1 EN cleavage site was then calculated.

The above analyses were performed on the loci with the candidate L1s in the sense orientation. In order to investigate the possibility that the candidate L1s were inserted in the antisense orientation, both microhomology and EN cleavage site analyses were repeated on the reverse complements of our sequences. In these cases, the 5' junctions closest to the poly(T) stretches were analysed as if they were 3' poly(A) stretches.

## Abbreviations

EN: endonuclease; LINE: long interspersed element; LTR: long terminal repeat; NHEJ: non-homologous end joining; ORF: open reading frame; PCR: polymerase chain reaction; RT: reverse transcriptase; TPRT: target primed reverse transcription; TSD: target site duplication; UTR: untranslated region.

## Competing interests

The authors declare that they have no competing interests.

## Authors' contributions

TJM, DS and MAB designed the research; TJM, DS and EMC performed the research; MAB contributed new reagents/analytic tools; TJM, DS and EMC analysed the data; and TJM, DS and MAB wrote the paper.
